# Healthy and sustainable development of sports economy based on artificial intelligence and mental model

**DOI:** 10.3389/fpsyg.2022.956682

**Published:** 2022-07-28

**Authors:** Yue Liu, Bo Dong, Xiangcheng Zeng

**Affiliations:** ^1^Sports Institute, Liaoning Normal University, Dalian, China; ^2^College of Physical Education and Health, LinYi University, Linyi, China; ^3^Physical Education Department, Dongbei University of Finance and Economics, Dalian, China

**Keywords:** sports economy, healthy and sustainable development, deep learning network, support vector machine, mental model

## Abstract

In recent years, sports have achieved rapid development worldwide, and the global economy has been significantly improved and improved. With the in-depth development of the two, the connection between sports and the economy has also become closer. Sports economy is a new type of economic form bred by specialization of sports organization, participation in consumerization, and profit-oriented operation under the condition of market economy. And the development of sports economy cannot be developed at once; it needs healthy and sustainable development. In order to find a better way to study the healthy and sustainable development of sports economy, this paper uses deep learning network algorithm and supports vector machine learning algorithm to build a mental model. It then uses the model to analyze various indicators of the sports industry in a province in China. This article is looking for information and summarizes the province’s sports data from 2017 to 2021. The sports indicators of this experiment include regional GDP, total output of sports industry, sports practitioners, local financial sports expenditures, the number of policies, the number of people participating in physical exercise, and fitness venues and facilities. The realization results show that these variables develop at a relatively small rate under normal conditions, and then predict the data in the next few years under the healthy and sustainable development of the next few years through the mental model. The growth rates of various indicators of the sports economy have increased significantly, and they have been optimized by about 20% compared with the normal development.

## Introduction

Sport is a social and cultural practice that has evolved with human evolution. Simple physical movement has become an important part of sociocultural practice, and even an integral part of people’s daily culture. Today, the whole human society is researching and practicing the concept of sustainable development. As part of general social practice, sport must also fit into the larger social family. It uses the theory of sustainable development to support and guide its own development. Otherwise, we can only go upstream, not only will it not develop well, but it will also become an obstacle to the development of the entire society. And when the level of economic development reaches a certain level, it will lay the foundation for the development of the sports industry. Therefore, it has important theoretical and practical significance to analyze the healthy and sustainable development of sports economy. It is equally important to find a good mental model to study the development of sports economy.

In order to have a deeper understanding of the relationship between sports and the economy, researchers have done a lot of research on it. Among them, Wei studied the relationship between the sports industry and economic development, and built a related index system. He found a positive correlation between the stability of the sports industry and economic development (Wei et al., 2017). Research of Wu took Xi’an as the research object. He analyzed the carrying capacity of water resources and its existing problems by constructing an evaluation system, and put forward countermeasures and suggestions for the development of water sports (Wu, 2018). Huang analyzed the current situation of China’s sports market economy development, and proposed a comprehensive evaluation model for the development capability of sports market economy. He specifically described the development path of the sports market economy (Huang and Wang, 2017). Yelamos studied sport in line with the sustainable development agenda, proposing a new perspective on health and well-being that is aligned with the broader interdisciplinary agenda (Yelamos et al., 2019). Sapkota explored grassroots development and peace (SDP) organizations in Nepal and found that NGOs related to “Youth and Sport” contribute to the SDGs in a number of ways. It shows that sports have a positive impact on economic development and peacebuilding (Sapkota and Neupane, 2021). But they did not have a good method when they were researching.

So, this has led some researchers to study the research method. Among them, Li studied some typical algorithms used in the development history of robot motion planning, and compared the performance of the algorithms, which played an important role in the development of artificial intelligence in sports (Li, 2017a). Huang studied the urban modern architectural art based on artificial intelligence and GIS image recognition system, and studied in detail the application of artificial intelligence systems in all aspects of intelligent construction (Huang et al., 2021). Chen H studied innovation and entrepreneurship based on artificial intelligence systems and neural network algorithms. He improved the algorithm according to the actual needs (Chen, 2021). Weerd used Bayesian models and mental models to determine the extent to which people use the Theory of Mental Reasoning in specific competitive games (Weerd et al., 2018). Based on the development trend of artificial intelligence technology, Cong studied the application of artificial intelligence in college sports information services (Cong and Fu, 2020). But there are some problems with their approach.

This paper builds a mental model based on artificial intelligence algorithms to study the healthy and sustainable development of the sports economy, and analyzes the changes in various indices of the sports industry. The innovation of this paper is that this paper constructs an optimal mental model under the deep learning algorithm and support vector machine algorithm. This paper uses the model to analyze the sports-related data of a province in China from 2017 to 2021, and then forecasts the data for the next few years. And this article is to analyze the variables of the sports industry development of the province’s sports industry and the variables that measure the economic development.

## Artificial intelligence and mental models

### Artificial intelligence

**Figure 1:**
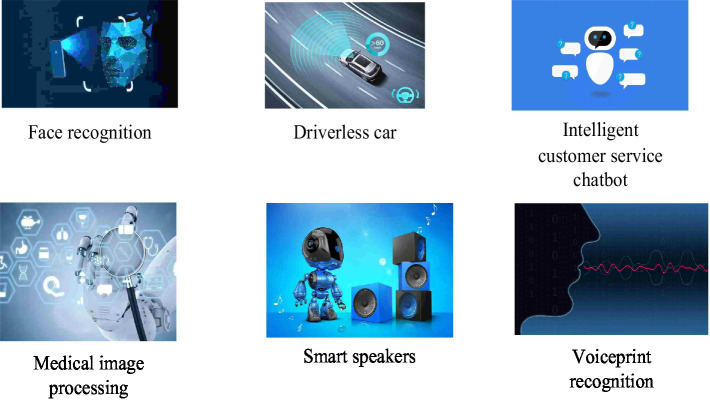
Artificial intelligence application areas.

Artificial intelligence (AI) is an important branch of computer science. It mainly expands the theory and technology of human intelligence and applied systems through research, development, and exploration. It is a comprehensive edge science that simulates human intelligence activities (Li, 2017b). It seeks to understand the nature of intelligence and produce a new type of intelligent machine that responds in a similar way to human intelligence. Research in the field of artificial intelligence is shown in Figure 1. Its fields include face recognition, driverless cars, intelligent customer service chatbots, medical image processing, voiceprint recognition, smart speakers, etc.

**Figure 2:**
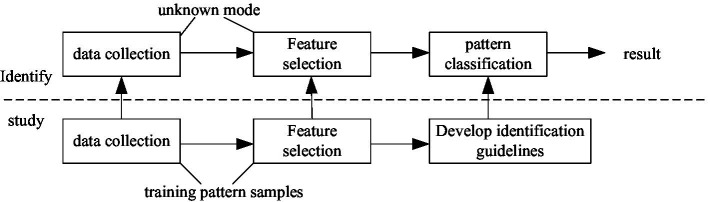
Pattern recognition process.

The most important part of artificial intelligence is the pattern recognition part. Because this part contains the algorithm of artificial intelligence, the recognition method can be classified according to the different algorithm objects (Li and Chen, 2018). Its specific process is shown in Figure 2.

### Artificial intelligence algorithms

Deep learning originated from the further study of artificial neural network algorithms. The deep neural network algorithm mainly combines the lower-level features skillfully to form higher-level feature information that is more abstractly expressed, in order to further discover the distributed feature information expression in the original data set. Deep learning has achieved many results in search technology, data mining, machine learning, machine translation, natural language processing, multimedia learning, speech, recommendation and personalization technology, and other related fields. It appears mainly to develop neural network algorithms that can imitate the mechanism of the human brain to store, understand, and interpret large amounts of data in the real world (Lu et al., 2017).

**Figure 3:**
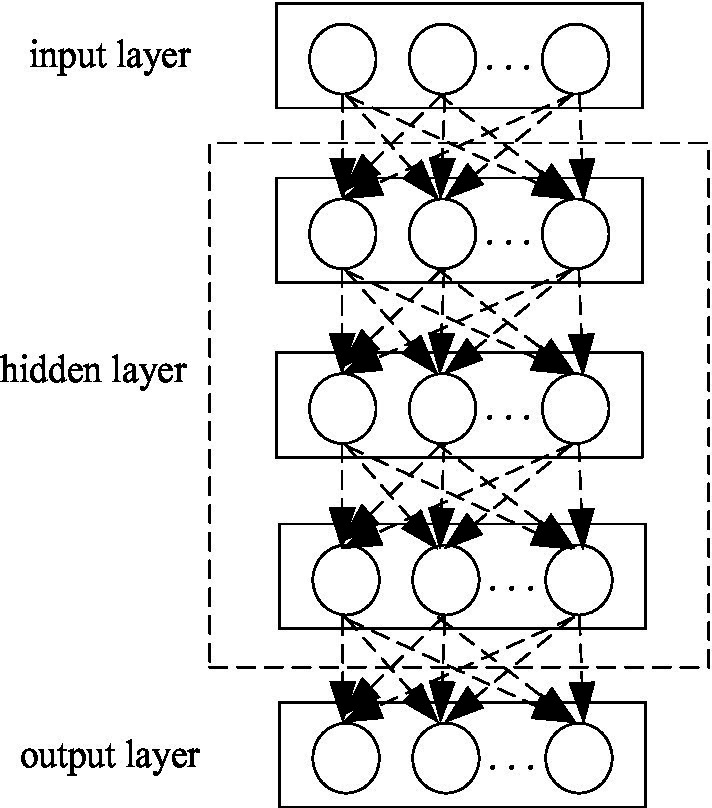
Deep learning network model.

This is equivalent to that if the human brain has strong memory analysis ability, a large number of connections between neurons must be required in the brain (Guo and Shang, 2017). Figure 3 vividly gives a schematic diagram of the deep learning network model in the general sense.

As shown in Figure 3, we can see that the deep learning network model can be regarded as an artificial neural network model with multiple hidden layers in essence. Deep neural network algorithms can perform systematic learning of nonlinear mapping structures on training datasets. The feature information of all data sample points in the training data set can be shared and learned by the same multi-layer neural network structure. It also further enhances the memory capability of the deep neural network algorithm model. The reason why the deep learning model has a strong learning ability of nonlinear mapping. The main reason is that the excitation function in the neuron node of each hidden layer is nonlinear, which makes the input value and output value of each hidden layer neuron node have a nonlinear representation relationship. In such a deep learning model, the difference in learning strategies of elements such as weight coefficients and activation functions between each two layers determines the type of deep learning model. The main deep learning model types are RBM model structure, DBN model structure, and CNN model structure, etc. (Favreau et al., 2017).

Among them, RBM is a model of energy minimization theory, which is a class of stochastic neural network models with two-layer structure, symmetrical connections, and no self-feedback. The layers are fully connected, and there is no connection within the layer. And its probability model can be expressed as:


(1) $$P\left(\mathrm{x,h}\right)=\frac{e^{E\left(\mathrm{x,h}\right)}}{Z}$$

(2) $$Z=\underset{x,h}{\sum }e^{-E\left(\mathrm{x,h}\right)}$$

**Figure 4:**
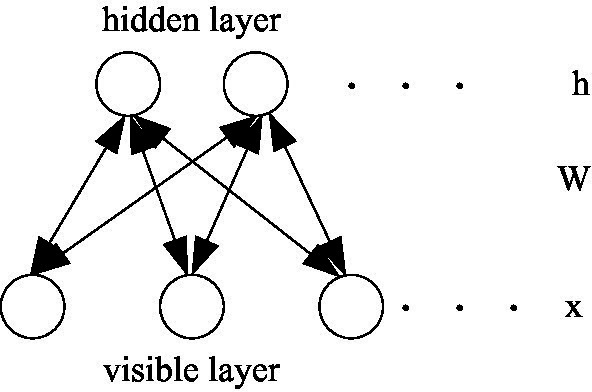
Schematic diagram of RBM.

Among them, $E\left(\mathrm{x,h}\right)$ represents the energy contained in the RBM model, and *x* and *h* represent the variables of the visible layer and the hidden layer in the RBM, respectively, as shown in Figure 4.

As shown in Figure 4, the same layers of the RBM model are independent of each other, so the energy function of the RBM model can be expressed as:


(3) $$E\left(\mathrm{x,h}\right)=-b^{T}x-c^{T}h-h^{T}Wx$$

Among them, b^T^, c^T^ and h^T^ represent the model parameters, hidden layer and visible layer between the two layers, respectively. When the state of each node in the visible layer is determined, we can deduce that the activation states of each hidden node are conditionally independent.


(4) $$P\left(h|x\right)=\Pi_{i}P\left(h_{i}|x\right)$$

Generally speaking *h*_i_ ∈ {0,1}, then the activation probability of the ith node of the hidden layer can be expressed as:


(5) $$P\left(h_{i}|\;x\right)=\mathrm{sigm}\left(c_{i}+W_{i}x\right)$$

Since the two variables *x* and *h* are symmetric in the energy function formula (3), the same can be obtained:


(6) $$P\left(x\;|\;h\right)=\underset{i}{\prod }P\left(x_{i}|h\right)$$

and


(7) $$P\left(x_{i}\;|\;h\right)=\mathrm{sigm}\left(b_{i}+W_{j}^{T}h\right)$$

*W_j_* represents the first column of the matrix.

Support vector machine (SVM) is a predictive algorithm model that is used to deal with two-class linear classification problems (Harahap and Kartika, 2020). SVM can also perform nonlinear classification through the kernel method, which is one of the common kernel learning methods. It is different from traditional predictive algorithm models. The foundation of the support vector machine algorithm is not the principle of empirical risk minimization, but is based on the minimization of structural risk.

For a dataset that is linearly separable in the feature space, there may be a large or even infinite number of separation hyperplanes in the corresponding feature space, and these separation hyperplanes can perfectly separate the two types of data completely. The formula corresponding to the separating hyperplane here is


(8) w⋅x+b=0

**Figure 5:**
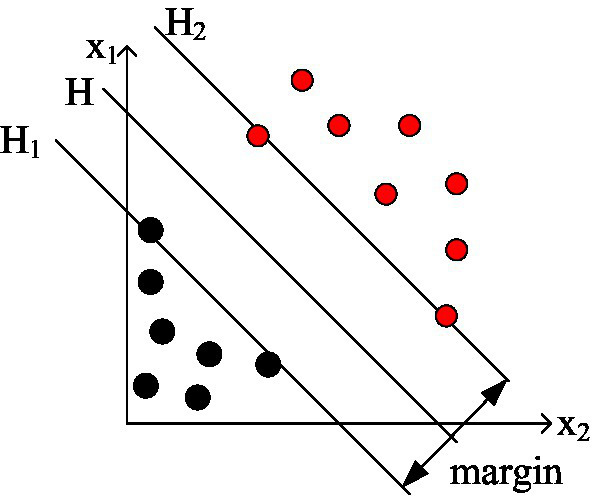
Optimal separating hyperplane.

The learning goal of SVM is to find an optimal separating hyperplane. In any linearly separable feature space, there is only one such perfect optimal separation hyperplane that can completely separate the training data by category. Such an optimal separation hyperplane can maximize the two-class classification interval (Zhang and Zhang, 2017; Huo et al., 2021), such as any linearly separable two-class classification learning problem on a two-dimensional plane, as shown in Figure 5.

As shown in Figure 5, we use black solid points and red solid points to represent positive sample points and negative sample points, respectively. The H line can be represented as the optimal separation line that separates the two types of data exactly and correctly (which can be mapped to the optimal separation hyperplane in high dimensions). The two straight lines, respectively, represent the classification boundary lines that pass through all the sample points in the positive sample point set and the negative sample point set that are closest to the optimal separation line and remain parallel to the optimal separation line. According to the above description, we can define the classification margin as the distance between the two straight lines. It is also denoted as a classification interval. The so-called optimal classification line is the line that can perfectly separate all the sample points of the two types of data in the training data set under the feature space and maximize the classification interval.

In order to maximize the separation interval, it is necessary to ensure that there is a good normal vector W that minimizes the value of *x*. At the same time, this good normal vector also needs to make the separating hyperplane can correctly classify all the sample points in the training data set.


(9) $$l_{i}\left(w\cdot x_{i}+b\right)-1\geq 0,i=1,2,\ldots .,N$$

In order to solve this unique optimal separating hyperplane, we can solve the optimization problem of the following linearly separable SVM learning.


$$\min_{w,b}\frac{1}{2}∥w{∥}^{2}$$

(10) $$s.t.l_{i}\left(w\cdot x_{i}+b\right)-1\geq 0.i=1,2,\ldots ,N$$

We only need to obtain the optimal solution w^*^, b^*^ of the constrained optimization problem, and then we must be able to obtain the optimal separation hyperplane and classification decision function expressed by the optimal solution.


(11) $$w^{*}\cdot x+b^{*}=0$$

(12) $$f\left(x\right)=\mathrm{sign}\left(w^{*}\cdot x+b^{*}\right)$$

It can also be said to obtain a linearly separable support vector machine model.

Then construct the Lagrangian function: introducing the corresponding Lagrangian multiplier term *α = (α_1_,α_2_,...,α_N_)^T^* to the inequality constraints corresponding to all the sample points in the training data set under the feature space, and then define the following Lagrangian function:


(13) $$L\left(w,b,a\right)-\frac{1}{2}||w||{}^{2}-\overset{N}{\underset{i=1}{\sum }}\alpha_{i}l_{i}\left(w\cdot x_{i}+b\right)+\overset{N}{\underset{i=1}{\sum }}\alpha_{i}$$

Among them, *α = (α_1_,α_2_,...,α_N_)^T^* represents a numerical vector composed of Lagrange multipliers corresponding to all sample points. Then, the above-mentioned Lagrangian function is obtained by partial differentiation of wL(w, b, a) and bL(w, b, a)respectively, and the value of the formula after partial differentiation is set equal to zero, which is expressed as follows:


(14) $$\nabla_{w}L\left(w,b,a\right)=0\Rightarrow w=\overset{N}{\underset{i=1}{\sum }}\alpha_{i}l_{i}x_{\begin{array}{c} i \end{array}}$$

(15) $$\nabla_{b}L\left(w,b,a\right)=0\Rightarrow \overset{N}{\underset{i=1}{\sum }}\alpha_{i}l_{i}=0$$

Then after simplification, we can get


(16) $$L\left(w,b,a\right)=\overset{N}{\underset{i=1}{\sum }}\alpha_{i}-\frac{1}{2}\overset{N}{\underset{i=1}{\sum }}\overset{N}{\underset{j=1}{\sum }}\alpha_{i}\alpha_{j}y_{i}y_{j}\left(x_{i}\cdot x_{j}\right)$$

After the above transformation, the convex optimization problem can be transformed into the corresponding Lagrangian dual problem, and the optimal Lagrangian term coefficient vector *α = (α_1_,α_2_,...,α_N_)^T^* can be obtained by solving the Lagrangian dual optimization problem, then


(17) $$w^{*}=\overset{N}{\underset{i=1}{\sum }}\alpha_{i}^{*}l_{i}x_{i}$$

(18) $$b^{*}=l_{j}-\overset{N}{\underset{i=1}{\sum }}\alpha_{i}^{*}l_{i}\left(x_{i}\cdot x_{j}\right)$$

### Mental model

Mental Model is the user’s existing knowledge and experience of the behavior and concept of the product. It also includes the user’s expectation when using the product, which comes from the user’s long-term life and the experience gained from using related products, as well as the personal thinking mode of cognition of things (Naess, 2019; Perna et al., 2019). It is also a comparative description or representation constructed to explain the process of human internal mental activity. It describes and elucidates a mental process or event. The construction of mental models is often accompanied by the construction of two other models, one of which is the Implementation Model. It is the developer’s perception of how the product is organized and deployed, including its internal structure and how it works. Another is the Represented Model. It is an understanding of how a product is used and works through observation and uses after the final product is designed and presented (Haleem et al., 2022; Lv and Guo, 2022).

**Figure 6:**
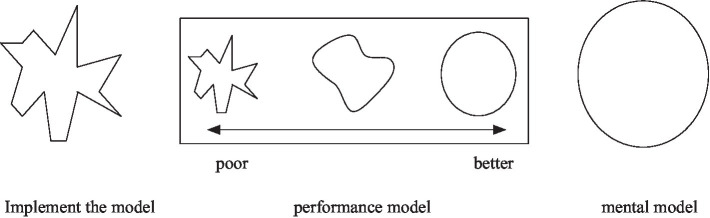
Relationship between the three models.

In general, the more complex the system, the greater the difference between the mental model and the implementation model, and vice versa. The performance model is the image model after the designer integrates the first two. The closer the performance model is to the user’s mental model, the easier the product is to understand and use, as shown in Figure 6.

**Figure 7:**
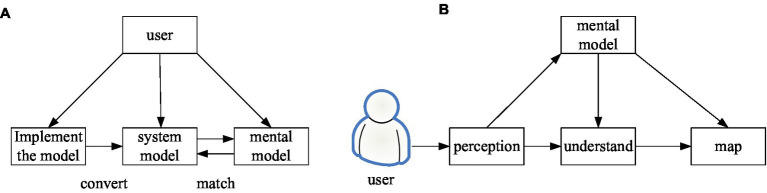
Construction of the mental model **(A,B)**.

Mental models cannot be abstracted. However, we can explore the common psychological characteristics of users through research on specific groups, and model them through certain events to obtain the psychological model of specific users. First of all, from the user’s point of view, the difference in cognitive structure lies in the difference in its knowledge structure, and the difference in knowledge structure lies in the difference in the groups to which it belongs. Second, in essence, the user’s “experience that already exists in his head” and “the newly acquired experience when using a new product” exist in the user’s cognition of the use of the product. There are two common mental model building modes shown in Figure 7.

In this way, the analysis of the mental model is guided to the decomposition of the user group and the analysis of the usage requirements of the product scenario. That is to say, taking a relevant description of the needs of a certain group of users to use the product in their product usage scenarios means a preliminary grasp of the mental model. As shown in Figure 7, in the construction of the mental model, the first thing we need to determine is the user group. The user group shows group characteristics and also shows universality. However, the mental model tends to be more personalized, and only the personalized bias has the refined meaning of scene shaping. That is, the refinement of user groups is not a large group classification, but the shaping of specific user roles based on user groups. User research is an approach to personas. It divides some user groups with significantly different behaviors, attitudes, goals, motivations, etc., through qualitative research and quantitative research, and extracts the personalization among them. It shapes it into a character, in anticipation of placing it in certain scenes to gain a rich and detailed experience with the product. The processing of the scene also decomposes the entire large event into detailed scenes through the analysis and refinement of the scene. In these scenarios, user personas’ descriptions of requirements also begin to be refined and personalized. Through such a mental model composed of characters, scenes, and goals to define products, the shaped products are more in line with the psychological expectations of users.

## Experiments on the healthy and sustainable development of sports economy

### Sports economy and sustainable development

Sports economy refers to the development of a unique industry that organically integrates the sports life of the public and related business activities from the perspective of production and management. The sports industry refers to the industry that supplies various sports labor products to the whole society with living labor, and it is all production and operation activities related to sports. The important function of its products is to improve the physical quality of residents, develop social production, stimulate the national spirit, and realize the all-round development of individuals and the all-round progress of social civilization. The sports industry can be divided into three categories, as shown in Table 1.

**Table 1:** Sports industry classification.

Sports industry	Sports ontology industry	Sports competition
		Physical fitness
		Sports training
	Sports related industries	Sports lottery
		Sporting goods
		Sports ads
	Sports industry	Repast
		Hotel
		Air ticket agent

As shown in Table 1, the sports industry is mainly divided into sports body industry, sports-related industry, and sports industry. The ontology industry mainly includes sports competitions, sports fitness, sports training, and other content. Related industries mainly include sports advertising, sports lottery, sports goods, and other content. The sports industry mainly includes catering, hotels, air ticketing, etc.

**Figure 8:**
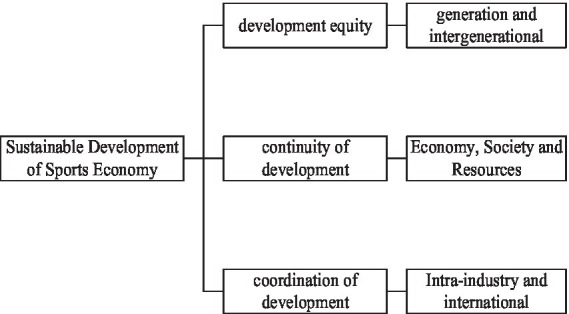
Relationship between principles of sustainable development of sports economy.

In a word, sustainable development is a kind of development based on the mutual coordination and common development of society, economy, population, resources, and environment. The sustainable development of sports economy also adheres to the principles of fairness, continuity, and coordination. The relationship between them is shown in Figure 8.

It can be seen from Figure 8 that the goal of sustainable development of sports economy is fairness, continuity, and coordination of development. Equity is a fundamental goal of sustainable development. The so-called fairness refers to the choice of equal opportunities, and its connotation is very rich. In the sustainable development of sports, it mainly includes two aspects: one is the fairness provided by sports within the generation, that is, the horizontal fairness between modern people. The sustainable development of sport must meet the basic sporting needs of all people. It provides sporting rights and opportunities for all to achieve their aspirations for a better quality of life. Second, the fairness of intergenerational sports resources, that is, vertical justice between current and future generations.

The law of sustainable development is basically a law that can be explained by duration, which reflects the dynamic and long-term nature of the sustainable development of the system. Sustainability refers to the ability of a system to develop in a stable and orderly manner. The resources and environment are the material basis and necessary conditions for people’s survival and development. The sustainable development of sports can promote the continuous increase of sports management investment, which is also the basic guarantee for improving the people’s sports technical level and survival quality.

Coordination includes interdepartmental coordination as well as international coordination. This reflects the multi-factor and complexity of the sustainable development of sports. Only when the population, society, economy, resources, environment, and other departments are in a state compatible with sports, human sports activities can continue to be carried out in an orderly manner. This coordination is also fully coordinated under the guidance of the principles of justice and continuity, not only to coordinate the different relationships between people, but also to coordinate the different relationships between people and places.

### Experiments on healthy and sustainable development

The research object of this experiment is the sports industry of a Province A in China and the changes in its sports economy. Through the query of relevant data, we know the scale of its sports industry in the past 5 years, as shown in Table 2.

**Table 2:** The scale of the sports industry in Province A in the past 5 years.

Years	Sports market size (100 million yuan)	Growth rate (%)
2017	553	–
2018	681	23.15
2019	803	17.91
2020	921	14.69
2021	1,035	12.38

As shown in Table 2, from 2017 to 2020, the scale of the sports market in Chinese province A has increased year by year, from 55.3 billion yuan in 2017 to 103.5 billion yuan. However, the growth rate of the province’s sports market size is decreasing year by year, and the rate of decrease of the annual growth rate is slowly getting smaller. This shows that the sports market in the province needs further development. In addition, this time, the variables related to the development of the sports industry in Province A from 2017 to 2021 and the variables that measure the economic development of Province A are selected, as shown in Table 3.

**Table 3:** Variable descriptive statistics.

Indicator classification	Variable	Mean
Economic indicators	Gross Regional Product (H/100 million yuan)	41021.64
	Total output of sports industry (I/100 million yuan)	814.94
Sports industry indicators	Sports practitioners (J/10,000 people)	7.74
	Local financial sports expenditure (K/100 million yuan)	107.37
	Number of policies (L/piece)	1.10
	Number of people participating in physical exercise (M/10,000 people)	2712.38
	Fitness venue facilities (N/piece)	6614.80

**Figure 9:**
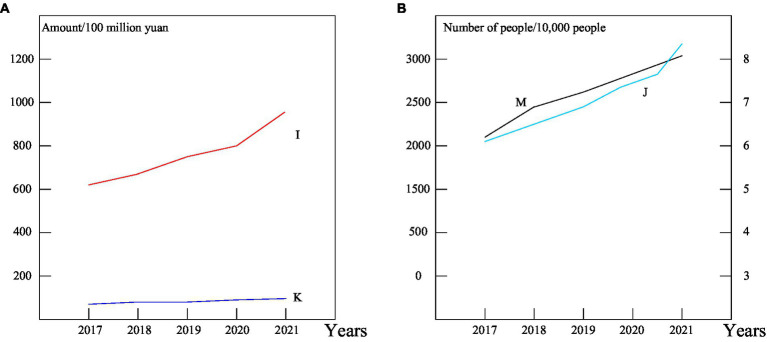
Development trend of relevant sports industry indicators **(A,B)**.

It can be seen from Table 3 that from 2017 to 2021, the average GDP of Province A is 4,102.164 billion yuan, and the average total output of the sports industry is 81.494 billion yuan. Under the sports industry indicator, the average number of sports practitioners is 77,400, and the average local financial expenditure on sports is 10.737 billion yuan. However, the average number of policies to develop the sports industry is less than 2, the average number of people who regularly participate in physical exercise is 27.1238 million, and the average number of fitness venues is 6614.80. And the changes between them are shown in Figure 9.

Figure 9A is a line graph showing the trend of the total output of the sports industry and the local fiscal expenditure on culture and sports from 2017 to 2021. As shown in figure, from 2017 to 2021, the total output of the sports industry in Province A has achieved rapid growth, and the total output of the sports industry in 2021 has exceeded 100 billion yuan. In recent years, the A provincial government has increased sports expenditure year by year, guided by the goal of building a strong sports province. It supports the optimization and upgrading of industries and improves the construction of industrial bases. The total output of the sports industry has grown rapidly for 5 consecutive years. A province’s local financial sports expenditure has increased year by year and maintained a relatively stable development trend. Figure 9B shows the changing trend of the number of sports practitioners and the number of regular exercisers in Province A from 2017 to 2021. According to the graph, from 2017 to 2021, the number of sports practitioners in province A has changed slightly, and the number of people who regularly participate in physical exercise has increased significantly.

**Figure 10:**
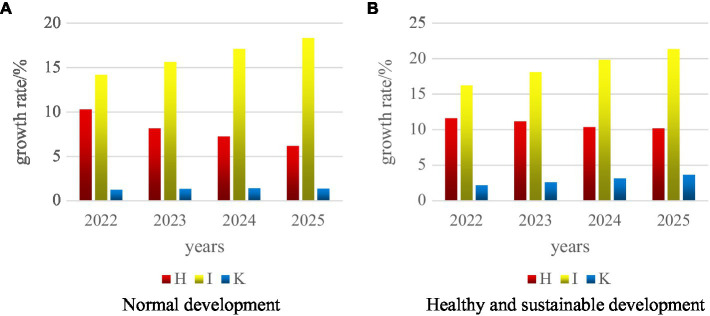
Prediction of H, I, and K three indicators. **(A)** Normal development. **(B)** Healthy and sustainable development.

In order to better study the development of sports economy, this experiment uses deep learning network algorithm and supports vector machine learning algorithm to build a mental model, and then predicts future development. This paper mainly analyzes and compares the growth rates of the indicators in Table 3. The forecast data is made on the basis of considering the healthy and sustainable development of the sports economy, and then compared with the growth of the sports economy under normal circumstances, as shown in Figure 10.

As shown in Figure 10, under normal circumstances, the development of province A’ growth rates of the three indexes of the total output of the sports industry and the local financial sports expenditure in the next few years are lower than those of the three indexes of sports development based on the healthy and sustainable development of the sports economy. Moreover, the annual growth rate is still relatively slow. In addition, the growth rate of the province’s GDP under normal development continues to decline. Although the GDP under the basis of healthy and sustainable development is also decreasing, the rate of decrease is relatively low. And the last predicted data are about 10%, and it may not change much after a longer period of time.

In addition, the number of sports practitioners, the number of people participating in physical exercise, and fitness facilities will continue to grow in the next few years, and the annual growth rate will also increase year by year. And this has also contributed to the growth of the GDP of Province A. Combining all the data, we can know that the healthy and sustainable development of sports economy has a great role in promoting the development of Province A.

### Recommendations

Vigorously increasing the number of people who regularly participate in exercise is conducive to the healthy and sustainable development of the sports economy. It is suggested to increase the number of people who regularly participate in physical exercise from the aspects of improving the awareness of physical exercise, construction of physical exercise venues, holding community sports events, popularization and guidance of physical exercise, publicity and channel construction of physical exercise, and promotion of physical exercise policies. Moreover, by increasing the number of people who regularly participate in physical exercise, it can promote the purchase of corresponding sporting goods, the viewing of sports events, and the reduction of medical and health risks, thereby promoting the sustainable development of the sports economy.

It is necessary to optimize the corresponding construction of sports industry professionals and increase the ability to attract sports industry professionals. The first is to strengthen the construction of sports industry talent protection policies, and implement preferential measures for sports industry talents in the fields of housing, loans, transportation, and medical care. The second is to strengthen school-enterprise cooperation, encourage multi-party joint efforts to cultivate corresponding talents in the sports industry, and improve employment and internship opportunities and job security. The third is to build an industry-university-research base for the sports industry. Through the discussion and practice of talent training programs, employment programs, incentive systems, exchanges, and mutual assistance in key areas of the sports industry, it forms the rear pillar for the formation of talents in the sports industry. At the same time, it should also encourage and support relevant personnel to engage in the sports industry and create a good employment environment.

## Conclusion

This paper mainly analyzes the data related to the sports industry in a certain province in China in recent years and conducts a direct analysis. It then builds a mental model based on these data, and then analyzes the growth trend of the province’s sports-related parameters in the next few years on the basis of the healthy and sustainable development of the sports economy. This paper also gives corresponding development suggestions. However, there are still some deficiencies in this paper. This paper only analyzes the sports economic data in a certain area, and does not analyze and compare multiple objects. But on the whole, the data used this time are specific sports economic data, which is very convincing. And with the continuous development of the sports industry, there are more and more studies on the healthy and sustainable development of the sports economy, thereby promoting the development of society. Moreover, people will further study the sustainable development of the sports industry, so that the development of the sports industry is more in line with the development concept of the society.

## Data availability statement

The original contributions presented in the study are included in the article/supplementary material; further inquiries can be directed to the corresponding author.

## Author contributions

YL: writing–original draft preparation. BD and XZ: editing data curation and supervision. All authors contributed to the article and approved the submitted version.

## Conflict of interest

The authors declare that the research was conducted in the absence of any commercial or financial relationships that could be construed as a potential conflict of interest.

## Publisher’s note

All claims expressed in this article are solely those of the authors and do not necessarily represent those of their affiliated organizations, or those of the publisher, the editors and the reviewers. Any product that may be evaluated in this article, or claim that may be made by its manufacturer, is not guaranteed or endorsed by the publisher.
